# Unicompartmental knee arthroplasty is associated with lower pain levels but inferior range of motion, compared with high tibial osteotomy: a systematic overview of meta-analyses

**DOI:** 10.1186/s13018-022-03319-7

**Published:** 2022-09-24

**Authors:** Hangyu Ping, Jiaxin Wen, Yubo Liu, Haifeng Li, Xin Wang, Xiangpeng Kong, Wei Chai

**Affiliations:** 1 School of Medicine, Nankai University, Tianjin, 300071 China; 2 Senior Department of Orthopedics, The Fourth Medical Center of PLA General Hospital, No.51 Fucheng Road, Haidian, Beijing, 100048 China; 3 National Clinical Research Center for Orthopedics, Sports Medicine & Rehabilitation, Beijing, 100853 China

**Keywords:** Unicompartmental knee arthroplasty, High tibial osteotomy, Osteoarthritis, Arthroplasty, Systematic review

## Abstract

**Background:**

The purpose of this study was to overview the findings of reported meta-analyses on unicompartmental knee arthroplasty (UKA) and high tibial osteotomy (HTO).

**Methods:**

The Preferred Reporting Items for Systematic Reviews and Meta-Analysis 2020 (PRISMA 2020) guidelines were followed. Two independent reviewers conducted a literature search of PubMed, Embase, the Web of Science, and the Cochrane Database of Systematic Reviews for meta-analyses comparing UKA and HTO that were published prior to September 2021. Literature screening, data extraction, and article quality appraisal were performed according to the study protocol registered online at PROSPERO (CRD42021279152).

**Results:**

A total of 10 meta-analyses were identified, and different studies reported different results. Five of the seven meta-analyses showed that the proportion of subjects with excellent or good functional results was higher for UKA than for HTO. All three meta-analyses showed that UKA was associated with lower pain levels, and all six of the studies that included an analysis of range of motion (ROM) reported that UKA was inferior to HTO. Four of the eight meta-analyses found that total complication rates were lower for UKA. Only 3 of the 10 meta-analyses found that UKA had lower revision rates. Moreover, in the subgroup analysis, the revision and complication rates of UKA were similar to those of opening-wedge HTO but much lower than those of closing-wedge HTO.

**Conclusions:**

Compared to HTO, UKA was associated with lower pain levels but inferior postoperative ROM. The results were inconclusive regarding whether UKA yielded better knee function scores and lower revision or complication rates than HTO. Accurate identification of indications and appropriate patient selection are essential for treating individuals with OA.

**Supplementary Information:**

The online version contains supplementary material available at 10.1186/s13018-022-03319-7.

## Background

Osteoarthritis (OA) of the knee is a common degenerative joint disease worldwide. In many patients, arthritic changes occur primarily in the medial compartment of the knee joint [1].

For medial knee OA, unicompartmental knee arthroplasty (UKA) and high tibial osteotomy (HTO) are well-established therapeutic options. A number of clinical studies and meta-analyses have compared UKA and HTO but have yielded inconsistent conclusions [2–7]. Therefore, a systematic overview is required to review the findings of reported meta-analyses and compare the outcomes between the two surgical methods.

The present study was performed to investigate, summarize, and critically appraise the findings of meta-analyses on UKA and HTO (e.g., clinical and functional outcomes and complication and revision rates) to aid clinical decision-making.

## Materials and methods

This overview of meta-analyses followed the guidelines for Preferred Reporting Items for Systematic Reviews and Meta-Analysis 2020 (PRISMA 2020) [8]. The protocol for this study was registered at PROSPERO (CRD42021279152).

### Search strategy

The following established medical databases were searched separately by two independent authors: PubMed, Embase, Web of Science, and the Cochrane Database of Systematic Reviews. The search was performed to identify relevant studies published prior to September 2021, with no language restrictions, using the following key terms: “knee,” “osteoarthritis,” “unicompartmental,” “arthroplasty,” “tibia*,” “osteotomy,” and “meta-analysis.” The search strategy is presented in detail in Supplementary Materials.

The titles/abstracts of the articles were then separately assessed to determine whether they met the criteria listed below. When conflicts arose, a third reviewer was consulted to obtain a consensus.

### Inclusion and exclusion criteria

The criteria for article inclusion were as follows:

- Original meta-analyses.
- Analyses of studies comparing UKA and HTO for medial knee OA.
- Studies with the following outcomes were included:
  o Functional scores, such as the Lysholm Knee Score (Lysholm), Knee Society Score (KSS), range of motion (ROM), and proportion of patients with excellent/good functional results.
  p Complication rates (e.g., for infection or thrombosis).
  q Implant survival and revision rates, such as Kaplan–Meier analysis of revision rates and implant survival time to total knee arthroplasty (TKA).

Studies that met the following criteria were excluded:

- Data were reported only for the UKA or HTO group, and no comparisons were made between the two groups.
- Duplicate reports.

For studies that met the preliminary eligibility criteria, full texts were retrieved. When differences arose, a third reviewer evaluated the different judgments to discuss and resolve contradictions. Any relevant studies that may have been missed were checked in the reference lists for more eligible publications.

### Data extraction

Two authors independently extracted data from the eligible meta-analyses. The data extracted were as follows: study details (author and year of publication); search details (follow-up, number and type of studies included); appraisal tool used; and analysis details (method of analysis, pooled outcomes recorded by more than two included studies, heterogeneity, and findings). The findings were compared, and any disparities were discussed until a consensus was achieved. Regarding the data of primary clinical literature in the meta-analysis meeting the inclusion criteria, relevant data extraction was performed according to the needs of the study.

### Study quality assessment

A Measurement Tool to Assess Systematic Reviews-2 (AMSTAR-2) [9] was used by two authors independently to evaluate the methodological quality of the included reviews. The AMSTAR-2 assesses review quality across 16 categories; seven of the elements are regarded as crucial, and weaknesses in any of these critical categories might affect the overall validity of a study. For the primary clinical literature in meta-analyses, we assessed the risk of bias in the nonrandomized studies using the Risk of Bias in Non-Randomized Studies of Interventions (ROBINS-I) assessment tool [10]. If any conflicts arose, a third reviewer was consulted to reach a final consensus.

### Interpretation of results

We tabulated and narratively summarized the findings from the eligible meta-analyses. To minimize bias, outcomes reported by less than three studies were not included. Outcomes are reported as the mean difference (MD), standard mean difference (SMD), risk ratio (RR), or odds ratio (OR), and *P* < 0.05 was taken to indicate statistical significance.

OR is used to describe the dichotomized variable (yes/no) for further quantitative synthesis. Random effects models fitted to a restricted maximum likelihood (REML) model were performed for evaluating the difference between UKA and different types of HTO. To assess the association between revision rates and publication years, a meta-regression analysis model was used with publication year as the independent variable and LogOR for revision rate as the dependent variable. STATA 16.0 was used for statistical analysis.

## Results

### Open literature search

**Fig. 1:**
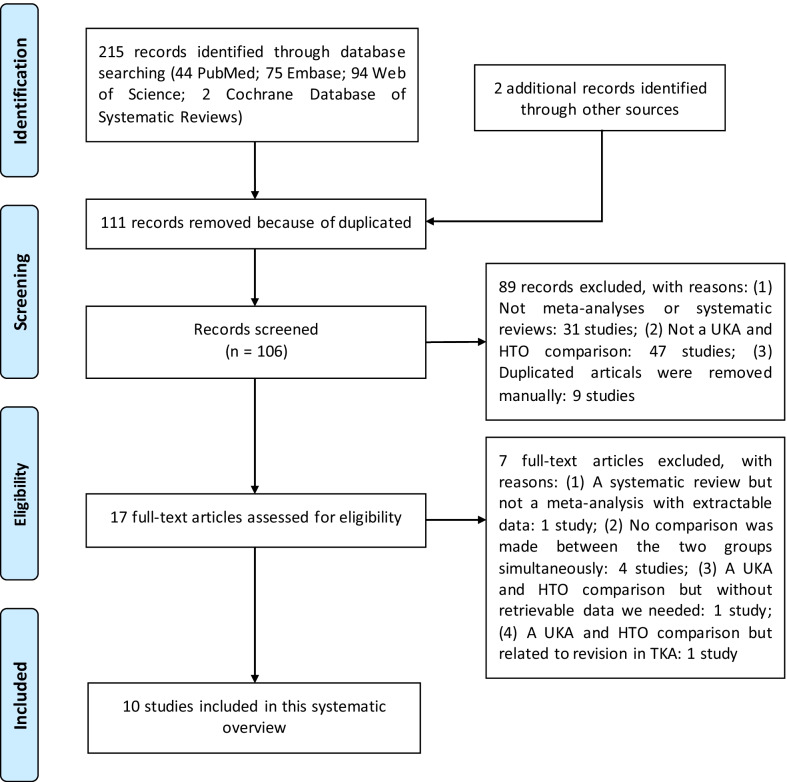
Study selection process and reasons for exclusion

The search retrieved 216 studies, of which 110 were duplicates. After screening of the titles and abstracts, 17 studies qualified for full-text screening, and 7 articles [2, 11–15] were excluded. Ultimately, a total of 10 meta-analyses, which were published between 2009 and 2020, were eligible for data extraction [3–7, 16–21]. The flowchart of the study selection process and the reasons for exclusion are presented in Fig. 1. The study characteristics are shown in Table 1, and outcomes for each study are shown in Table 2.

**Table 1:** Characteristics of the meta-analyses on UKA versus HTO

Author /Year	Number of studies assessed	Number of knees (UKA/HTO)	Follow-up, months (range)	Outcomes reported by ≥ 3 studies						Number of original studies	HTO type, number of studies
				Functional results	ROM	Velocity	Pain	Revision rate	Complication rate		
Migliorini et al. [7]	RCT 1 Retrospective 6	311/307	24–90	Yes				Yes		7	OW 7 CW 0
Bai et al. [35]	RCT 2 Retrospective 11	332/379	12–120	Yes	Yes			Yes	Yes	13	NA
Huang et al. [17]	Prospective 1 Retrospective 7	304/371	30–84	Yes	Yes	Yes		Yes	Yes	8	OW 8 CW 0
Cao et al. [3]	RCT 2 Retrospective 7 Register Study 1	5305/868	0–90	Yes	Yes		Yes	Yes	Yes	10	OW 6 CW 3
Santoso et al. [5]	RCT 3 Prospective 2 Retrospective 9 Register Study 1	5497/1041	0–204	Yes	Yes	Yes	Yes	Yes	Yes	15	OW-HTO 6 CW-HTO 7
Han et al. [19]	RCT 4 Prospective 3 Retrospective 9	603/591	0–204	Yes	Yes	Yes	Yes	Yes	Yes	16	OW 5 CW 11
Fu et al. [21]	RCT 3 Prospective 2 Retrospective 5 Register Study 1	5081/759	0–204	Yes	Yes	Yes		Yes	Yes	11	OW-HTO 3 CW-HTO 7
Spahn et al. [6]	UKA 40 HTO 43 Comparative Studies 3	4742/4090	60–240	Yes				Yes	Yes	93	OW-HTO 4 CW-HTO 38
Zhang et al. [16]	RCT 3 Prospective 1 Retrospective 3	196/219	12–204	Yes				Yes	Yes	7	OW 1 CW 5
Gandhi et al. [20]	RCT 3 Prospective 1 Retrospective 2	186/176	6–93.6	Yes		Yes			Yes	6	OW 1 CW 5

*NA* Not available

**Table 2:** Clinical outcomes for each study

Author/Year	Clinical and functional results					Revision rates					Complications rates				
	Effect size [95% CI]		*P* value	*n*^a^	For	Effect size [95% CI]		*P* value	*n*^a^	For	Effect size [95% CI]		*P* value	*n*^a^	For
Migliorini et al. [7]	Tegner	MD 0.69 [0.03–1.35]	.04		UKA	5 years revision rate	OR 2.27 [0.50–10.34]	.004		UKA					
	Lysholm	MD 3.07 [1.19–4.95]	.001		UKA	Kaplan‒Meier curve		.01		UKA					
	IKDC	MD 8.89 [4.29–13.48]	.0001		UKA										
	KOOS	MD 2.27 [− 4.23–8.77]	.05		UKA										
Bai et al. [35]	Tegner	MD 0.00 [-0.18–0.18]	.97	5	n.s	Revision rate	OR 1.17 [0.48–2.82]	.73	4	n.s	Total Complications rate	OR 0.51 [0.24–1.07]	0.07	9	n. s
	Lysholm	MD 0.84 [0.29–1.39]	.003	6	UKA						Infection rate	OR 0.91 [0.24–3.50]	0.89	4	n. s
	E/G results	OR 1.34 [0.49–3.67]	.57	4	n.s										
	ROM	MD − 5.47 [− 9.53 to − 1.41]	.008	7	HTO										
Huang et al. [17]	HSS	MD 1.25 [− 3.70–1.20]	.32	3	n.s	Revision rate	OR 0.38 [0.07–1.89]	.23	4	n.s	Total Complications rate	OR 1.11 [0.49–12.49]	0.81	7	n.s
	Lysholm	MD − 0.90 [− 3.70–1.89]	.53	2	n.s										
	ROM	MD 10.18 [2.49–17.86]	.009	3	HTO										
	velocity	MD 0.02 [− 0.09–0.04]	.49	3	n.s										
Cao et al. [3]	Lysholm	MD 4.99 [− 3.91–13.09]	.27	2	n.s	Revision rate	OR 0.52 [0.30–0.90]	.02	8	UKA	Complications (total)	OR 0.42 [0.20–0.89]	0.02	5	UKA
	KSS	MD − 4.03 [− 9.91–1.85]	.18	2	n.s						Complications (RCT)	OR 0.20 [0.04–1.00]	0.05	1	UKA
	E/G results	OR 2.18 [0.58–8.23]	.25	4	n.s						Complications (n-RCT)	OR 0.54 [0.22–1.30]	0.17	4	n.s
	ROM	SMD − 0.85[− 1.43 to − 0.27]	0.04	4	HTO										
	Pain	OR 5.65 [1.24–25.81]	.03	2	UKA										
Santoso et al. [5]	Knee scores ^b^	STD − 0.21 [− 0.47–0.05]	0.11	7	n.s	Revision rate	OR 1.18 [0.54–2.58]	0.68	11	n.s	Complications rate	OR 3.08 [1.76–5.39]	< 0.0001	7	UKA
	E/G results	OR 0.37 [0.24–0.58]	< 0.00001	10	UKA										
	Subgroup: E/G CW-HTO-UKA	OR 0.36 [0.21–0.61]	0.01	6	UKA										
	Subgroup: E/G OW-HTO-UKA	OR 0.70 [0.26–1.91]	0.49	3	n.s										
	ROM	SMD 0.78 [0.21, 1.36]	0.008	5	HTO										
	Velocity	SMD − 0.09 [− 0.48, 0.30]	0.66	3	n.s										
	Pain	OR 0.34 [0.13, 0.91]	0.03	5	UKA										
Han et al. [19]	E/G results	OR 0.47 [0.24–0.95]	0.04	10	UKA	Revision–TKA (total)	OR 1.56 [0.61–3.98]	0.35	7	n.s	Complications rate	OR 2.48 [1.26 to 4.90]	0.009	8	UKA
	ROM	MD 8.62 [2.02–15.23]	0.01	6	HTO	Subgroup: CW-HTO vs. UKA	OR 2.38 [1.05–5.42]	0.04	4	UKA					
	Pain	OR 0.28 [0.12–0.62]	0.002	4	UKA	Subgroup: OW-HTO vs. UKA	OR 0.24 [0.03–2.00]	0.19	3	n.s					
	Velocity	MD − 0.05 [− 0.11 to − 0.00]	0.03	4	UKA										
Fu et al. [21]	Knee scores^c^	SMD 0.78 [− 0.75, 2.30]	0.32	4	n.s	Revision rate	OR 0.82 [0.30–2.21]	0.69	7	n.s	Complication rate	OR 2.00 [0.62, 6.50]	0.25	4	n.s
	E/G results	OR 0.43 [0.26, 0.69]	0.0006	8	UKA										
	ROM	SMD 1.36 [1.05, 1.67]	< 0.00001	5	HTO										
	Velocity	SMD − 0.49 [− 0.98, 0.01]	0.05	3	UKA										
Spahn et al. [6] Fu et al. [21]	normalized knee scores^d^				Survival to endpoint TKA rate				Complication rate						
	5–8 years of follow-up	HTO 83.4 [82.6–84.2]	< 0.001	7	UKA	5–8 years of follow-up	HTO 0.910 [0.882–0.932]	0.801	30	n.s	HTO 0.138 [0.107, 0.177]	UKA 0.113 [0.079, 0.168]	0.369	UKA 31/HTO 13	n.s
		UKA 91.2 [90.9–91.4]		5			UKA 0.915 [0.882–0.939]		26						
	9–12 years of follow-up	HTO 79.9 [76.9, 82.8]	< 0.001	7	UKA	9–12 years of follow-up	HTO 0.844 [0.797–0.882]	0.458	28	n.s					
		UKA 90.0 [89.7, 90.2]		5			UKA 0.869 [0.814–0.909]		25						
	After more than 12 years of follow-up	HTO 58.8 [47.6, 69.9]	0.331	2	n.s	More than 12 years of follow-up	HTO 0.701 [0.605–0.782]	0.451	15	n.s					
		UKA 65.6 [57.4, 73.9]		3			UKA 0.775 [0.583–0.895]		9						
	Knee score from baseline to 5–8 years of follow-up	HTO SMD 5.0 [3.2–6.8]	0.359	7	n.s	Mean survival (Kaplan–Maier)	HTO 9.7 years [8.1–11.2]	0.374	12	n.s					
		UKA SMD 4.1 [3.2–4.7]		3			UKA 8.2 years [5.5–11.0]		5						
	Knee score from baseline to 9–12 years of follow-up	HTO 1.7 [1.0, 2.3]	< 0.001	8	UKA										
		UKA 10.7 [10.2, 11.1]		1											
	Score from baseline to more than 12 years of follow-up	HTO -0.2 [− 0.5, 0.1]	0.603	1	n.s										
		UKA 1.2 [0.7, 1.6]		1											
Zhang et al. [16]	E/G result	OR 2.43 [1.46, 4.05]	0.0006	6	UKA	Revision rate	OR 0.47 [0.23–0.97]	0.04	5	UKA	Complication rate	OR 0.24 [0.10, 0.56]	0.001	4	UKA
Gandhi et al. [20]	E/G result	OR 2.03 [1.16–3.6]	0.013	6	UKA	survival from aseptic loosening	OR 2.14 [0.93–4.93]	0.074	5	n.s					
	Velocity	SMD 0.389 [− 0.124–0.902]	0.137	2	n.s										

*UKA* Unicompartmental knee arthroplasty, *HTO* High tibial osteotomy, *n.s.* Not significant, *CI* Confidence interval, *SMD* Standard mean difference, *MD* Mean difference, *RR* Relative risk, *OR* Odds ratio, *E/G result* Excellent and good functional results, *Tegner* Tegner activity scale, *Lysholm* Lysholm Knee Score, *KSS* Knee Society Score, *IKDC* International Knee Documentation Committee, *KOOS* Knee Injury and Osteoarthritis Outcome Score, *HSS* Hospital for Special Surgery Score, *ROM* range of motion

^a^Number of studies reporting on an outcome

^b^Knee scores included the Baily Knee Score, Knee Society Score, British Orthopaedic Association Score, and Hospital for Special Surgery Score

^c^Knee scores included the Lysholm Knee Score and the Knee Society Score

^d^Each study used a self-created 100-point score

### Methodological quality

Five articles [3–5, 16, 21] were rated as “moderate” quality, and three [7, 17, 20] were rated as “low” quality. In addition, two papers [6, 18] were of “critically low” quality, meaning that they failed to fulfill four of the seven critical items. (See the details in Table 3.)

**Table 3:** Evaluation of the quality of meta-analyses using the AMSTAR-2 quality assessment tool

Item no	1	2 *	3	4 *	5	6	7 *	8	9 *	10	11*	12	13*	14	15*	16	Overall rating
Migliorini et al. [7]	√	×	×	P	√	√	×	√	×	×	√	√	√	×	√	√	Low
Bai et al. [35]	√	×	×	P	×	×	×	√	√	×	√	×	×	√	×	√	Critically low
Huang et al. [17]	√	×	×	P	√	√	×	√	√	×	√	√	√	√	×	√	Low
Cao et al. [3]	√	√	×	P	√	√	×	√	√	×	√	√	√	√	√	√	Moderate
Santoso et al. [5]	√	×	×	P	√	√	×	√	√	×	√	√	√	√	√	√	Moderate
Han et al. [19]	√	√	×	P	√	√	×	√	√	×	√	√	√	√	√	√	Moderate
Fu et al. [21]	√	×	×	P	√	√	×	√	√	×	√	√	√	√	√	√	Moderate
Spahn et al. [6]	√	×	×	P	√	√	×	P	×	×	√	√	√	√	×	×	Critically low
Zhang et al. [16]	√	×	×	P	√	√	×	√	√	×	√	√	√	√	√	√	Moderate
Gandhi et al. [20]	√	×	×	P	√	√	×	√	√	×	√	√	√	√	×	√	Low

Item 1: described the inclusion for PICO. Item 2: registered the protocol of the review before it was conducted. Item 3: considered the reasons for inclusion of the studies. Item 4: had a comprehensive search strategy. Item 5: completed the study selection independently. Item 6: conducted the data extraction independently. Item 7: provided a list of excluded studies with reasons. Item 8: described the characteristics of the included studies in detail. Item 9: used appropriate tools to assess the risk of bias in the included studies. Item 10: reported the funding sources. Item 11: used proper methods for this meta-analysis. Item 12: discussed the potential risk of bias in the included studies. Item 13: considered the risk of bias in interpreting the results. Item 14: discussed heterogeneity. Item 15: discussed publication bias. Item 16: disclosed funding or conflicts of interest

* Critical domains in AMSTAR-2

**√** clear documentation that the study met this itemized requirement, P unclear evidence from the article, × no evidence that requirements were met

### Clinical and functional results

#### Functional results

Seven studies [3, 5, 16, 18–21] reported the proportions of subjects with excellent or good functional results (E/G results). Five of these studies [5, 16, 19–21] reported that the rate of E/G results was higher for patients undergoing UKA than HTO, while the other two studies [3, 18] reported no difference between the two patient groups (Table 2).

Seven studies [3, 5–7, 17, 18, 21] used various scoring systems to compare knee scores between UKA and HTO (Table 2). The Lysholm Knee Score is commonly reported as a subjective measure of patients’ day-to-day knee function and general condition [22]. Of the four studies [3, 7, 17, 18] that reported Lysholm scores, two [7, 18] reported better scores for UKA than for HTO (MD = 3.07, 95% confidence interval [CI] = 1.19–4.95; MD = 0.84, 95% CI = 0.29–1.39), while the remaining two studies [3, 17] found no difference between the two methods. Three studies [5, 6, 21] used the SMD as the effect size to allow a meta-analysis of different scoring systems, and one study [6] reported that the normalized knee score was significantly better after UKA than after HTO over a 5–12-year follow-up period (*P* < 0.001); however, the scores of the two groups were comparable after more than 12 years (*P* = 0.331). There were no significant differences in the other two studies [5, 21], although scores tended to be higher for UKA than for HTO (Table 2).

#### Range of motion

Six studies [3, 5, 17–19, 21] included an analysis of ROM, and all found that HTO was better than UKA for this parameter (SMD = 0.78–1.36, WMD = 5.47–10.18) (Table 2).

#### Velocity

Of the five studies that assessed postoperative velocity [5, 6, 17, 19, 21], four [5, 6, 17, 21] showed no significant differences between groups. The remaining study [19] indicated that the UKA group tended to reach a faster velocity (WMD =  − 0.05, 95% CI − 0.11 to − 0.00) (Table 2).

#### Pain assessment

All three studies [3, 5, 19] that included postoperative pain assessments reported lower postoperative pain in the UKA group (Table 2).

### Revision surgery

All 10 studies [3, 5–7, 16–21] compared revision rates between UKA and HTO. Seven of the studies [5, 6, 17–21] reported similar revision rates between UKA and HTO, while the other three [3, 7, 16] found that the revision rates were lower and the implant survival time was longer in UKA than HTO (Table 2).

One study [19] found no difference in revision rate between UKA and HTO (OR = 1.56, *P* = 0.35). After performing a subgroup analysis of patients treated with HTO, the revision rate of the opening-wedge HTO (OW-HTO) subgroup was similar to that of the UKA group (*P* = 0.19), while the closing-wedge (CW-HTO) subgroups had a significantly higher revision rate than the UKA group (OR = 2.38, *P* = 0.04). One study [6] analyzed the implant survival time to TKA and the revision rates for UKA and HTO. The mean implant survival time (according to the Kaplan‒Meier method) to revision was 8.2 years in the UKA group and 9.7 years in the HTO group. The difference in revision rate between UKA and HTO after more than 12 years of follow-up was not significant (Table 2).

### Complications

Eight studies [3, 5, 6, 16–19, 21] compared total complication rates between UKA and HTO. Four studies [6, 17, 18, 21] reported no difference in complication rate between UKA and HTO, while the remaining four studies [3, 5, 16, 19] indicated that the complication rates were lower in UKA (Table 2).

### Quantitative analysis

**Fig. 2:**
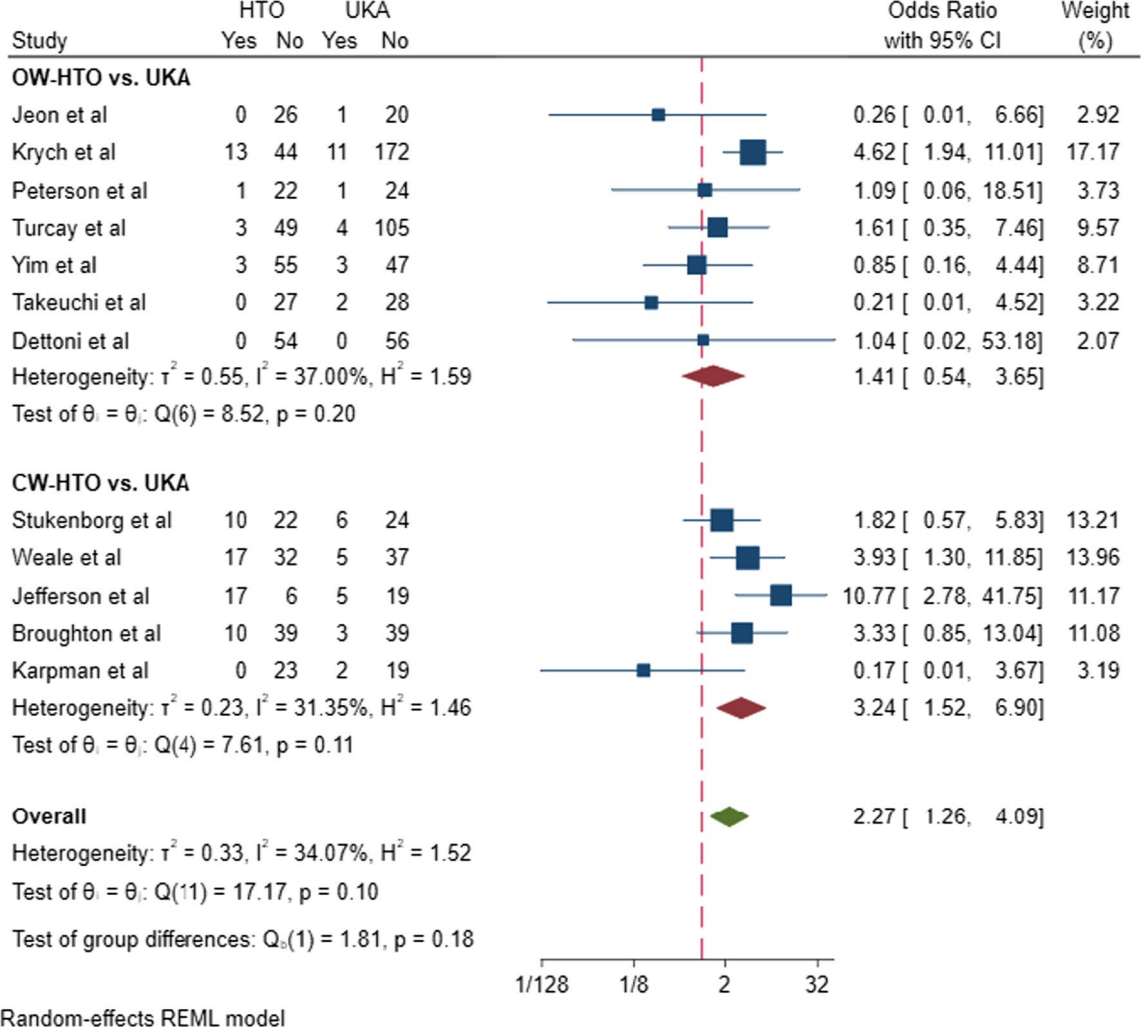
Revision rates in the subgroup analysis of OW-HTO versus UKA and CW-HTO versus UKA

**Fig. 3:**
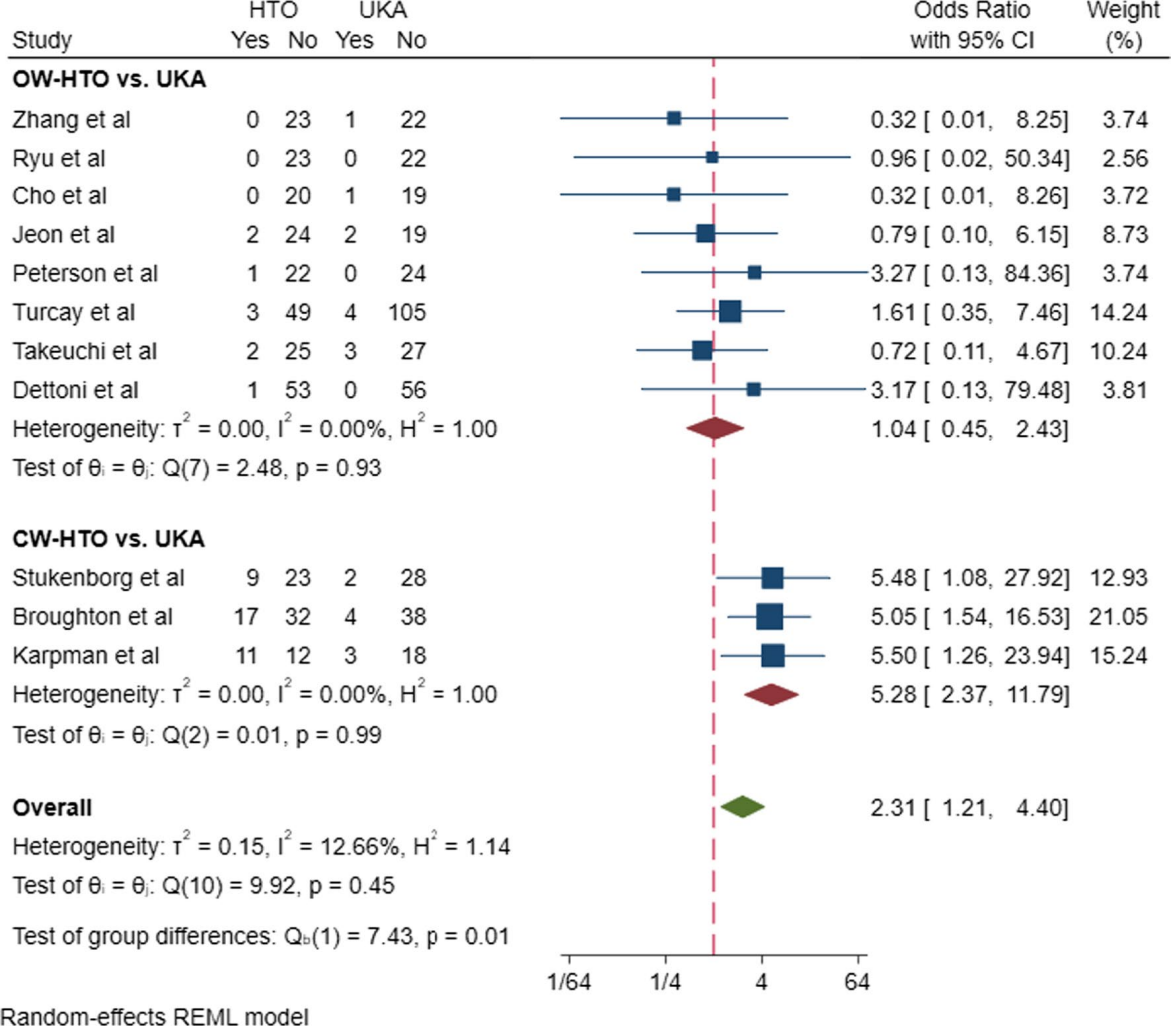
Complication rates in the subgroup analysis of OW-HTO versus UKA and CW-HTO versus UKA

**Fig. 4:**
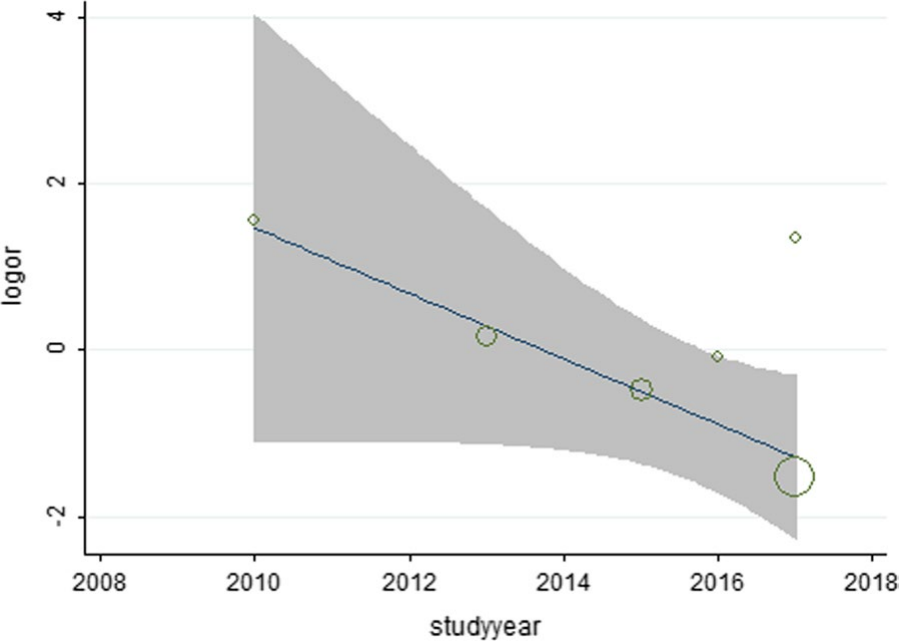
Meta-regression of revision rate and publication year for OW-HTO versus UKA

To gain insight into the complication and revision rates of UKA and HTO, we extracted and quantitatively synthesized the data from primary clinical studies included in all meta-analyses. A total of 20 studies were extracted [23–42]. (The study characteristics are shown in Additional file 1: Table 1, and the methodological quality assessment is shown in Additional file 1: Table 2.) The revision and complication rates were quantitatively synthesized based on the methodological quality assessment. The subgroup analyses were performed by types of HTO (OW-HTO or CW-HTO). In subgroup analyses, compared with CW-HTO, OW-HTO showed a lower revision rate (OW-HTO: OR = 0.71, 95% CI [0.27–1.85]; CW-HTO: OR = 0.31, 95%CI [0.14–0.66], Fig. 2) and a lower complication rate (OW-HTO: OR = 0.96, 95% CI [0.41–2.24]; CW-HTO: OR = 0.19, 95%CI [0.08–0.42], Fig. 3). Subsequently, a meta-regression analysis was performed based on HTO types (OW-HTO or CW-HTO), study types (prospective or retrospective study), and publication years, and HTO types were not found to be the source of heterogeneity in revision rate (*P* = 0.491) and complication rate (*P* = 0.845). However, a meta-regression analysis of OW-HTO group based on the publication year revealed that the postoperative revision rate of UKA decreased gradually with increase in year, and the predicted revision rate of UKA was lower than that of OW-HTO after 2014 (Fig. 4).

## Discussion

This review of meta-analyses showed that although UKA was associated with lower pain levels, compared to HTO, it also showed inferior postoperative ROM. The results were inconclusive regarding whether UKA had better knee function scores or lower revision and complication rates. Moreover, in the subgroup analysis, the revision and complication rates of UKA were similar to those of OW-HTO but much lower than those of CW-HTO.

### Clinical and functional results

This study showed that most meta-analyses (5 of 7) reported to date indicated that UKA is associated with better E/G results, and all three meta-analyses that examined postoperative pain assessments reported lower postoperative pain for UKA than for HTO. Moreover, all six meta-analyses that reported ROM showed that HTO achieved better outcomes. The discrepancy between clinical and functional outcomes suggests that additional factors may be involved. Immobilization and limited weight bearing for a certain period after HTO surgery may affect evaluations of postoperative function. Song et al. [43] followed up 60 HTO and 50 UKA patients for 20 years and found that the long-term survival rates of fixed platform implant UKA and CW-HTO were similar in groups with similar demographic characteristics and knee lesion severities, although the short-term clinical effects of UKA were better than those of HTO. Kim et al. [44] conducted a prospective study of 49 patients treated with HTO and 42 treated with UKA and reported that UKA was associated with superior VAS, WOMAC, and Lysholm scores at 3 and 6 months postoperatively, while the two procedures had similar scores at 1 year. Although there may be no significant difference in long-term outcomes between UKA and HTO, UKA tended to have superior postoperative outcomes in the short term, which is more consistent with the concept of enhanced recovery after surgery (ERAS).

All six meta-analyses examining ROM showed that HTO was associated with better postoperative ROM than UKA [3, 5, 17–19, 21]. However, among the primary clinical studies included in those meta-analyses, patients in the HTO groups tended to be younger and had a higher ROM than those in the UKA groups. Cao et al. [3] considered postoperative ROM to be dependent on the preoperative condition. Belsey et al. [2] conducted a systematic review regarding patients' return to physical exercise after HTO or UKA and discovered that patients who underwent HTO reached higher physical activity both pre- and postoperatively, while patients who underwent UKA exhibited a greater increase in physical exercise and superior function postoperatively. In addition, as there were no restrictions regarding arthroplasty components, the ROM was superior for HTO compared to UKA [19, 21]. Patients in the HTO groups tended to be younger, with milder arthritis and better preoperative function, although the degree of improvement in postoperative function tended to be smaller than that following UKA.

### Revision and complication rates

In our study, the results were inconclusive regarding whether the revision and complication rates were lower following UKA than they were after HTO. Based on the results of our subgroup analysis, OW-HTO may be a better option than CW-HTO in reducing revision and complication rate. In HTO cases, the most common cause of revision TKA was the degeneration of joint compartments. In UKA cases, there was the loosening of components, worn polyethylene inlays, damaged components, and postoperative pain [21]. Spahn et al. [6] conducted a meta-analysis and showed that UKA patients received TKA revision after an average of 8.2 years, compared to 9.7 years for those treated with HTO. Although the implant survival time was slightly shorter for UKA, there was a high degree of heterogeneity, and the results were not statistically significant. Several studies have reported that conversion to TKA following UKA was more difficult, and the revision rate after TKA was higher than that following HTO [14, 45–48]. El-Galaly et al. [45] analyzed 2,133 observations from the Danish Knee Arthroplasty Registry to conduct a propensity-score-weighted cohort study and found that the implant survival period until TKA following UKA was significantly shorter than that following HTO; the estimated 5-year implant survival rate was 0.88 for UKA (95% CI = 0.85–0.90) and 0.94 for HTO (95% CI = 0.93–0.96). Lee et al. [46] conducted a similar study of patients from the Korean National Health Insurance database who underwent TKA and found that for TKA after HTO, the risk of revision was lower than that for TKA after UKA, although there was no significant difference in complication rates following TKA after UKA versus after HTO. Lee et al. [14] conducted a meta-analysis and found that clinical outcomes (conversion to TKA) were similar for HTO and UKA, while conversion to TKA after UKA required more revision components and thicker polyethylene inserts. Lim et al. [47] reported the outcomes of UKA and HTO (i.e., revision TKA) at the 2-year follow-up and found that revision after UKA requires more revision components and an increased operation time but was associated with fewer complications than revision after HTO. Robertsson et al. [48] found that stemmed implants were used in revision to TKA in 4% (22 of 889) of cases after previous HTO and in 17% (136 of 920) of cases after UKA. In previous studies, the revision rate was lower for UKA than for HTO, but conversion to TKA after failed UKA was more difficult, and implant survival after revision to TKA was shorter for UKA than it was for HTO.

Complications of HTOs included neurovascular compromise, nonoptimal correction, nonunion, infection, implant complications, and cortical fracture, with reported incidences ranging from 5 to 36%. [49–52]. OW-HTO and CW-HTO are the most commonly used surgical techniques for HTO [3, 7]. According to a subgroup analysis of the primary clinical literature included in the meta-analyses, the complication rate of UKA was similar to that of OW-HTO but much lower than that of CW-HTO. Furthermore, based on the result of meta-regression, we found a trend of decreasing revision risk for UKA over time and it may be related to the maturity of UKA surgical techniques and the improvement of implant design. OW-HTO is considered a safer technique given the higher incidence of peroneal nerve paralysis associated with CW-HTO [5, 53], with reported rates of peroneal nerve injury ranging from 3.2 to 20% for CW-HTO [54–56]. Dorofeev et al. [49] reported that the overall complication rates were lower after OW-HTO than after CW-HTO (13.8% and 25.1%, respectively, *P* = 0.02), regardless of age or BMI. However, in a meta-analysis of randomized controlled trials (RCTs) comparing OW-HTO and CW-HTO, Wang et al. [57] found no significant differences in complication or implant survival rates between the two procedures, while OW-HTO increased the tibial slope angle. The discrepancies in results among studies suggest that additional factors may be involved, and further high-quality studies are needed to draw definitive conclusions.

### Indications

Patient selection is crucial to achieve good outcomes with both UKA and HTO. Previous literature reviews of UKA were based on classic indications [58–60], i.e., isolated medial or lateral knee compartment OA; age > 55 years; BMI ≤ 30 kg/m^2^; angular deformity < 15°; flexion contracture < 5°; ideal ROM > 90°; and joint stability. The surgical indications for UKA have been expanded to include younger and more active patients, commensurate with improvements in surgical techniques and implant designs. Plate et al. [61] analyzed 746 medial robot-assisted UKAs in patients with a mean BMI of 32.1 kg/m^2^ and reported that BMI did not influence the clinical results for robotic UKAs over a follow-up of more than 24 months. Hamilton et al. [62] compared the knee function of 449 patients weighing > 82 kg and 551 patients weighing < 82 kg after UKA and found no significant difference in American Knee Society Objective scores (AKSS-O), AKS Score-Functional (AKSS-F), or Oxford Knee Scores (OKS) at 10 years or implant survival at 15 years. Swienckowski et al. [63] reported an implant survival rate of 92% at 11 years in UKA patients younger than 60 years. A meta-analysis [64] showed that while younger patients had higher revision rates, they were more likely to achieve high postoperative functional scores. Therefore, age and obesity may not be justifiable as absolute contraindications to UKA.

For HTO, appropriate patient selection is equally essential to ensure the success of the surgery [53]. On the basis of results in the literature [65–68], ideal candidates for HTO are < 65 years of age and exhibit mild or moderate articular degeneration (≤ grade III from the Ahlback classification), isolated medial compartment OA, good ROM, and no ligamentous instability. Age and weight were once regarded as key factors in the selection of patients for HTO [69–74]. Akizuki et al. [73] reported that preoperative BMI > 27.5 kg/m^2^ and ROM < 100° were risk factors for early failure. Kanakamedala et al. [53] proposed that patients with BMI > 27.5 kg/m^2^ should be advised that their high BMI placed them at risk for worse pain relief and a higher risk of revision. In addition, Trieb et al. [75] reported that the relative risk rises 1.5 times in patients over 65 years old compared to younger patients.

In addition, some indications for HTO and UKA overlap [60, 76], i.e., age of 55–65 years, no joint instability, moderate activity ability, fair range of motion, mild varus alignment, and moderate arthrosis of the media compartment. Koh et al. [77] investigated preoperative factors associated with patient satisfaction following HTO and UKA with ideal patient selection criteria and suggested that a severe degree of OA was related to discontent after HTO, but dissatisfaction after UKA was related to young age and severe varus deformity. Smith et al. [11] evaluated the cost-effectiveness of UKA and HTO for treating medial knee OA patients of different ages and suggested that in younger patients, HTO may be the most cost-effective option; however, in elderly patients, UKA may be preferred.

Although the indications continue to expand, younger patients and those with extraarticular deformities may benefit more from HTO, while elderly patients with fewer activity demands or a more severe OA grade may be more suitable for UKA.

### Limitations

This review of meta-analyses had some limitations. First, the follow-up period varied greatly among studies. Second, RCTs are difficult to perform because of restrictions related to blinding and ethics. In most clinical studies, the final choice of operation was dependent on the decision of both the patient and surgeon. Therefore, it was difficult to ensure that the groups were balanced at baseline. Third, the AMSTAR-2 evaluation found that five of the included meta-analyses were of “low” or “critically low” quality, which limits the overall quality of evidence in our study. Fourth, the meta-analyses included in this study did not take the maturity of surgical techniques and modern implant designs into account. Fifth, some clinical studies were repeatedly included in many meta-analyses, which may have led to the superposition of some effects in our analysis. Finally, no meta-analyses evaluated the impact of the two different surgical approaches on health economics according to individual, health care provider, or clinical factors.

## Conclusion

UKA was associated with lower pain levels but inferior postoperative ROM compared to those of HTO. The results were inconclusive regarding whether UKA was associated with better knee function scores or lower revision or complication rates than HTO. Moreover, in the subgroup analysis, the revision and complication rates of UKA were similar to those of OW-HTO but much lower than those of CW-HTO.

Accurate identification of indications and appropriate patient selection are essential for all therapeutic approaches to OA. Age, BMI, grade of OA, and activity level should be taken into account during treatment planning. Finally, 5 of the 10 meta-analyses included in this overview were of “low” or “critically low” quality, and therefore, further well-designed large-scale clinical studies, high-quality systematic reviews, or meta-analyses are necessary to confirm our findings.

## Supplementary Information

**Additional file 1**. Chapter 1: Search strategy. Chapter 2: Additional Tables.

## Data Availability

Datasets are available through the corresponding author upon reasonable request.

## Abbreviations

UKA: Unicompartmental knee arthroplasty
HTO: High tibial osteotomy
TKA: Total knee arthroplasty
OW-HTO: Opening-wedge HTO
CW-HTO: Closing-wedge HTO
ROM: Range of motion
E/G results: Excellent or good functional results
VAS: Visual analogue scale
ERAS: Enhanced recovery after surgery
PRISMA: Preferred Reporting Items for Systematic Reviews and Meta-Analysis
AMSTAR-2: A Measurement Tool to Assess Systematic Reviews-2
MD: Mean difference
SMD: Standard mean difference
RR: Risk ratio
OR: Odds ratio
