# Supplementary material for: Unicompartmental knee arthroplasty is associated with lower pain levels but inferior range of motion, compared with high tibial osteotomy: a systematic overview of meta-analyses
Source: J Orthop Surg Res. 2022 Sep 24;17:425. doi: 10.1186/s13018-022-03319-7 (PMC9509560; doi:10.1186/s13018-022-03319-7)
Supplement: Supplementary file 1 — Additional file 1. Chapter 1: Search strategy. Chapter 2: Additional Tables. [file 13018_2022_3319_MOESM1_ESM.docx]

# Electronic Supplementary Material

## Chapter 1: Search strategy

***PubMed***

| Search number | Query | Results |
| --- | --- | --- |
| 9 | (#1 or #2) AND (#3 or #4) AND (#5 or #6) AND (#7 or #8) | 44 |
| 8 | (meta-analysis) OR ("Meta-Analysis as Topic"[Mesh]) OR ("Meta-Analysis" [Publication Type]) | 224,323 |
| 7 | (Systematic Review) OR ("Systematic Reviews as Topic"[Mesh]) OR ("Systematic Review" [Publication Type]) | 231,336 |
| 6 | ((Tibia*) AND ((Osteotomy) OR Osteotomies)) OR ((High tibial osteotomy)) | 7578 |
| 5 | "Osteotomy"[Mesh] | 37,150 |
| 4 | (((((((((((((((((((((((((Arthroplasties, Replacement, Knee) OR Arthroplasty, Knee Replacement) OR Knee Replacement Arthroplasties) OR Knee Replacement Arthroplasty) OR Replacement Arthroplasties, Knee) OR Knee Arthroplasty) OR Arthroplasty, Knee) OR Arthroplasties, Knee Replacement) OR Replacement Arthroplasty, Knee) OR Arthroplasty, Replacement, Partial Knee) OR Unicompartmental Knee Arthroplasty) OR Arthroplasty, Unicompartmental Knee) OR Knee Arthroplasty, Unicompartmental) OR Unicondylar Knee Arthroplasty) OR Arthroplasty, Unicondylar Knee) OR Knee Arthroplasty, Unicondylar) OR Partial Knee Arthroplasty) OR Arthroplasty, Partial Knee) OR Knee Arthroplasty, Partial) OR Unicondylar Knee Replacement) OR Knee Replacement, Unicondylar) OR Partial Knee Replacement) OR Knee Replacement, Partial) OR Unicompartmental Knee Replacement) OR Knee Replacement, Unicompartmental) | 41,311 |
| 3 | "Arthroplasty, Replacement, Knee"[Mesh] | 27,248 |
| 2 | (((Knee Osteoarthritides) OR (Knee Osteoarthritis)) OR (Osteoarthritis of Knee)) OR (Osteoarthritis of the Knee) | 42,000 |
| 1 | "Osteoarthritis, Knee"[Mesh] | 22,797 |

***Embase***

| No. | Query | Results |
| --- | --- | --- |
| #15 | #3 AND #6 AND #9 AND #14 | 75 |
| #14 | #10 OR #11 OR #12 OR #13 | 508,739 |
| #13 | 'systematic review':ab,kw,ti | 247,295 |
| #12 | 'systematic review'/exp | 312,969 |
| #11 | 'meta analysis':ab,kw,ti | 238,623 |
| #10 | 'meta analysis'/exp | 224,438 |
| #9 | #7 OR #8 | 565,658 |
| #8 | 'osteoarthritis'/exp | 144,482 |
| #7 | 'arthritis' OR 'arthrosis' OR 'osteoarthritis' OR 'osteoarthrosis':ab,kw,ti | 558,156 |
| #6 | #4 OR #5 | 59,351 |
| #5 | 'osteotom*':ab,kw,ti | 42,544 |
| #4 | 'osteotomy'/exp | 50,937 |
| #3 | #1 OR #2 | 54,546 |
| #2 | 'unicompartmental knee arthroplasty' OR 'unicondylar knee arthroplasty' OR 'knee arthroplasty' OR 'knee replacement' OR 'knee reconstruction' OR 'UKA':ab,kw,ti | 54546 |
| #1 | 'knee arthroplasty'/exp | 50323 |

***Web of Science***

| No. | Query | Results |
| --- | --- | --- |
| #5 | #1 AND #2 AND #3 AND #4 | 94 |
| #4 | **TS=((systematic review) or (meta analys*) or (meta-analys*))** | 635,390 |
| #3 | TS=((arthritis) OR (arthrosis) OR (osteoarthritis) OR (osteoarthrosis)) | 537,260 |
| #2 | **TS=(osteotom*)** | 59,950 |
| #1 | TS=((unicompartmental knee arthroplasty) OR (unicondylar knee arthroplasty) OR (knee arthroplasty) OR (knee replacement) OR (knee reconstruction) OR (UKA)) | 89,335 |

**Cochrane Database of Systematic Reviews**

| ID | Search | Results |
| --- | --- | --- |
| #1 | MeSH descriptor: [Arthroplasty, Replacement, Knee] explode all trees | 2699 |
| #2 | (arthroplasty or replacement or reconstruction):ti,ab,kw (Word variations have been searched) | 56736 |
| #3 | MeSH descriptor: [Osteotomy] explode all trees | 786 |
| #4 | (osteotom*):ti,ab,kw (Word variations have been searched) | 2140 |
| #5 | ( #1 or #2) and (#3 or #4) in Cochrane Reviews | 2 |

## Chapter 2: Additional Tables

***Table 1: Characteristics of the primary clinical studies in meta-analyses on UKA vs. HTO***

| Author | Year | Design | No | | UKA Model  /HTO Type | Follow-up | Complication | Revision | ROM | Function Score |
| --- | --- | --- | --- | --- | --- | --- | --- | --- | --- | --- |
| Chen et al | 2019 | Retrospective | UKA | 20 | OxfordⅢ | 1-3.2y | 0 | NA | NA | 91.5±1.7 (Lysholm scores) |
|  |  |  | HTO | 18 | OW-HTO | 1-2.8y | 0 | NA | NA | 90.6±2 (Lysholm scores) |
| Zhang et al | 2019 | Retrospective | UKA | 23 | NA | 0.26 | 1 | NA | NA | NA |
|  |  |  | HTO | 23 | OW-HTO | 0.28 | 0 | NA | NA | NA |
| Li et al | 2019 | Retrospective | UKA | 22 | OxfordⅢ | NA | NA | NA | 134.4±1.2 | 90.7±1.2 (Lysholm scores) |
|  |  |  | HTO | 18 | OW-HTO | NA | NA | NA | 134.9±1.6 | 90±1 (Lysholm scores) |
| Ryu et al | 2018 | Retrospective | UKA | 22 | Sigma unicompartmental Knee | 2.75 | 0 | NA | NA | 89.2±10 (Lysholm scores) |
|  |  |  | HTO | 23 | OW-HTO | 3.3 | 0 | NA | NA | 87.4±12 (Lysholm scores) |
| Cho et al | 2018 | Retrospective | UKA | 20 | Oxford knee | 3.3 | 1 | NA | 146.8±12.7 | 96.3±8.5 |
|  |  |  | HTO | 20 | OW-HTO | 4 | 0 | NA | 149.4±9.4 | 95.1±7.6 |
| Jeon et al | 2017 | Retrospective | UKA | 21 | Miller-Galante | 2 | 2 | 1 | NA | 104.04±20.64 (KOOS) |
|  |  |  | HTO | 26 | OW-HTO | 2 | 2 | 0 | NA | 103.92±20.91 (KOOS) |
| Krych et al | 2017 | Retrospective | UKA | 183 | Miller-Galante | 5.8 | NA | 11 | NA | 90.0±11.0 (Lysholm scores) |
|  |  |  | HTO | 57 | OW-HTO | 7.2 | NA | 13 | NA | 80.2±11.8 (Lysholm scores) |
| Peterson et al | 2016 | Retrospective | UKA | 25 | Oxford Ⅲ | 5 | 0 | 1 | NA | NA |
|  |  |  | HTO | 23 | OW-HTO | 5 | 1 | 1 | NA | NA |
| Turcay et al | 2015 | Retrospective | UKA | 109 | Oxford Ⅲ | 3.5 | 4 | 4 | NA | 90.00 (HSS) |
|  |  |  | HTO | 52 | OW-HTO | 3.4 | 3 | 3 | NA | 83.95 (HSS) |
| Karamitev et al | 2014 | Retrospective | UKA | 66 | NA | 4 | NA | NA | NA | NA |
|  |  |  | HTO | 103 | CW-HTO | 4 | NA | NA | NA | NA |
| Yim et al | 2013 | Retrospective | UKA | 50 | Miller-Galante | 3.7 | NA | 3 | 130±8.8 | 90.3±7.7 (LKS) |
|  |  |  | HTO | 58 | OW-HTO | 3.6 | NA | 3 | 138±4.7 | 89.6±8.7 (LKS) |
| W-Dahl et al | 2010 | register study | UKA | 4799 | NA | NA | NA | 816 | NA | NA |
|  |  |  | HTO | 450 | Hemicallotasis | NA | NA | 47 | NA | NA |
| Takeuchi et al | 2010 | Retrospective | UKA | 30 | Nakashima | 7 | 3 | 2 | 127±16 | 88±7.7 (KSS) |
|  |  |  | HTO | 27 | OW-HTO | 5.1 | 2 | 0 | 146±5.7 | 89±7.6 (KSS) |
| Dettoni et al | 2008 | Prospective | UKA | 56 | Accuris | 2-4y | 0 | 0 | NA | NA |
|  |  |  | HTO | 54 | OW-HTO | 2-4y | 1 | 0 | NA | NA |
| Borjeson et al | 2005 | RCT | UKA | 22 | Bragham | 5 | NA | NA | 123±0.5 | 37±2 (BOA) |
|  |  |  | HTO | 18 | CW-HTO | 5 | NA | NA | 123±0.5 | 37±0.75 (BOA) |
| Stukenborg et al | 2001 | RCT | UKA | 30 | Oxford/PCA | 7.5 | 2 | 6 | 103±26.25 | 66.5±9 (KSS) |
|  |  |  | HTO | 32 | CW-HTO | 7.5 | 9 | 10 | 117±12.5 | 73.5±5.9 (KSS) |
| Weale et al | 1994 | Retrospective | UKA | 42 | St Georg | 12-17y | NA | 5 | NA | NA |
|  |  |  | HTO | 49 | CW-HTO | 12-17y | NA | 17 | NA | NA |
| Weidenhielm et al | 1993 | RCT | UKA | 36 | Brigham | 1 | NA | NA | 119±14 | NA |
|  |  |  | HTO | 23 | CW-HTO | 1 | NA | NA | 121±9 | NA |
| Weidenhielm et al | 1992 | RCT | UKA | 28 | Brigham | 1 | NA | NA | NA | NA |
|  |  |  | HTO | 25 | CW-HTO | 1 | NA | NA | NA | NA |
| Ivarsson et al | 1991 | RCT | UKA | 10 | Oxford/PCA | 0.5 | NA | NA | 112±13 | 91±11 (Lysholm) |
|  |  |  | HTO | 10 | CW-HTO | 1 | NA | NA | 121±11 | 78±19 (Lysholm) |
| Jefferson et al | 1989 | prospective | UKA | 23 | Oxford/PCA | NA | NA | 17 | NA | NA |
|  |  |  | HTO | 24 | CW-HTO | NA | NA | 5 | NA | NA |
| Broughton et al | 1986 | Retrospective | UKA | 42 | St Georg | 5.8 y | 4 | 3 | NA | 39.6±7.3 |
|  |  |  | HTO | 49 | CW-HTO | 7.8 y | 17 | 10 | NA | 35.8±7 |
| Karpman et al | 1982 | Retrospective | UKA | 21 | NA | 3 | 3 | 2 | NA | NA |
|  |  |  | HTO | 23 | CW-HTO | 2 | 11 | 0 | NA | NA |

NA: not available

***Table 2: Methodological quality assessment (ROBINS-I).***

| Study | Year | Bias due to confounding | Bias in selection of participants | Bias in classification of interventions | Bias due to deviations from intended interventions | Bias due to missing data | Bias in measurement of outcomes | Bias in selection of the reported result | Overall Bias |
| --- | --- | --- | --- | --- | --- | --- | --- | --- | --- |
| Ryu et al | 2018 | Moderate | Moderate | Low | Low | Low | Low | Moderate | Moderate |
| Cho et al | 2018 | Low | Moderate | Low | Low | Low | Low | Moderate | Moderate |
| Jeon et al | 2017 | Low | Moderate | Low | Low | Moderate | Serious | Moderate | Moderate |
| Krych et al | 2017 | Low | Moderate | Low | Low | Low | Low | Moderate | Low |
| Petersen et al | 2016 | Moderate | Moderate | Low | Low | Moderate | Low | Moderate | Moderate |
| Tuncay et al | 2015 | Serious | Moderate | Low | Low | Moderate | Moderate | Moderate | Moderate |
| Karamitev et al | 2014 | Serious | Serious | Moderate | Moderate | Low | Low | Moderate | Moderate |
| Yim et al | 2013 | Moderate | Moderate | Low | Low | Low | Moderate | Moderate | Moderate |
| W-Dahl et al | 2010 | Serious | No information | No information | Low | Low | No information | Critical | Critical |
| Takeuchi et al | 2010 | Moderate | Moderate | Low | Low | Low | Moderate | Moderate | Moderate |
| Dettoni et al | 2008 | Moderate | Moderate | Moderate | Moderate | Low | Moderate | Moderate | Moderate |
| Borjesson et al | 2005 | Moderate | Low | Low | Moderate | Low | Low | Moderate | Low |
| Stukenborg et al | 2001 | Moderate | Low | Low | Low | Low | Low | Moderate | Low |
| Weale et al | 1994 | Moderate | Serious | Serious | Low | Low | Moderate | Moderate | Moderate |
| Weidenhielm et al | 1993 | Low | Moderate | Moderate | Low | Low | Moderate | Moderate | Moderate |
| Weidenhielm et al | 1992 | Low | Moderate | Serious | Low | Low | Low | Moderate | Moderate |
| Ivarsson et al^a^ | 1991 | - | - | - | - | - | - | - | - |
| Jefferson et al | 1989 | Low | Moderate | Low | Low | Low | Low | Moderate | Low |
| Broughton et al | 1986 | Moderate | Moderate | Moderate | Low | Low | Moderate | Moderate | Moderate |
| Karpman et al | 1982 | Moderate | Moderate | Serious | Moderate | Low | Low | Moderate | Moderate |

a: Full text not found
